# Celastrol inhibits mouse B16-F10 melanoma cell survival by regulating the PI3K/AKT/mTOR signaling pathway and repressing HIF-1α expression

**DOI:** 10.1007/s12672-024-01045-6

**Published:** 2024-05-21

**Authors:** Ping Zhao, Xing-Bo He, Xin-Yue Chen, Zhang-Long Li, Wen-Jia Xing, Wei Liu, Cong Ren, Xu-Dong Han, Bin Guo

**Affiliations:** 1 Medical College of Optometry and Ophthalmology, Shandong University of Traditional Chinese Medicine, Jinan, 250000 China; 2 School of Medicine, Southeast University, Nanjing, 210000 China; 3 College of Traditional Chinese Medicine, Shandong University of Traditional Chinese Medicine, Jinan, 250000 China

**Keywords:** Celastrol, B16-F10, PI3K/AKT/mTOR, HIF-α, ROS

## Abstract

**Objective:**

Melanoma, with its high degree of malignancy, stands as one of the most dangerous skin cancers and remains the primary cause of death from skin cancer. With studies demonstrating the potential of traditional Chinese medicine to intervene and treat melanoma, we turned our attention to celastrol. Celastrol is a triterpene compound extracted from the traditional Chinese medicine derived from *Tripterygium wilfordii*. Previous studies have shown that celastrol exerts inhibitory effects on various malignant tumors, including melanoma. Hence, our goal was to clarify the impact of celastrol on cell viability, apoptosis, and cell cycle progression by elucidating its effects on the PI3K/AKT/mTOR pathway.

**Methods:**

CCK-8 and wound healing assays were used to determine the effect of celastrol on the viability and migration of B16-F10 cells. Changes in cell apoptosis, cell cycle, reactive oxygen species (ROS), and mitochondrial membrane potential were detected by flow cytometry. PI3K/AKT/mTOR pathway proteins and HIF-α mRNA expression in B16-F10 cells were detected by western blotting and qPCR. Moreover, the addition of a PI3K activator demonstrated that celastrol could inhibit the function of B16-F10 cells via the PI3K/AKT/mTOR pathway.

**Results:**

Celastrol inhibited the viability and migration of B16-F10 cells. Through the inhibition of the PI3K/AKT/mTOR pathway down-regulates the expression of HIF-α mRNA, thereby causing an increase of ROS in cells and a decrease in the mitochondrial membrane potential to promote cell apoptosis and cell cycle arrest. The inhibitory effect of celastrol on B16-F10 cells was further demonstrated by co-culturing with a PI3K activator.

**Conclusion:**

Celastrol inhibits the function of B16-F10 cells by inhibiting the PI3K/AKT/mTOR cellular pathway and regulating the expression of downstream HIF-α mRNA.

## Introduction

Melanoma, a type of malignant tumor derived from melanocytes, is highly invasive, metastatic, and a major cause of skin cancer-related deaths [1, 2]. Melanocyte precursor cells are produced in the neural crest and migrate to the skin, mucous membranes, ocular uvea, pia mater, and other organs during embryonic development [3]. Ultraviolet (UV) radiation exposure and genetic factors have been identified as potential risk factors for melanoma [4, 5]. Although melanoma has a low incidence and mortality rate compared to breast cancer, lung cancer, and other cancers, advanced melanoma can spread to other organs, and the prognosis of patients is poor owing to its aggressiveness and metastasis [6–8].

Currently, melanoma can be treated with surgery, immunotherapy, targeted therapy, radiation therapy, chemotherapy, and other methods [9–12]. Although immunotherapy and targeted therapy can improve the progression-free and overall survival of patients, some patients with metastatic melanoma cannot respond to these therapies, and some patients are remarkably resistant to conventional cancer therapies or experience adverse side effects [13, 14]. Among various therapeutic means, drug therapy plays an important role in the treatment of tumors [15]. However, there is a lack of effective and safe drugs for the treatment of melanoma. Therefore, it is necessary to identify natural products with high efficiency and low toxicity for inhibiting the growth and metastasis of melanoma cells.

**Fig. 1:**
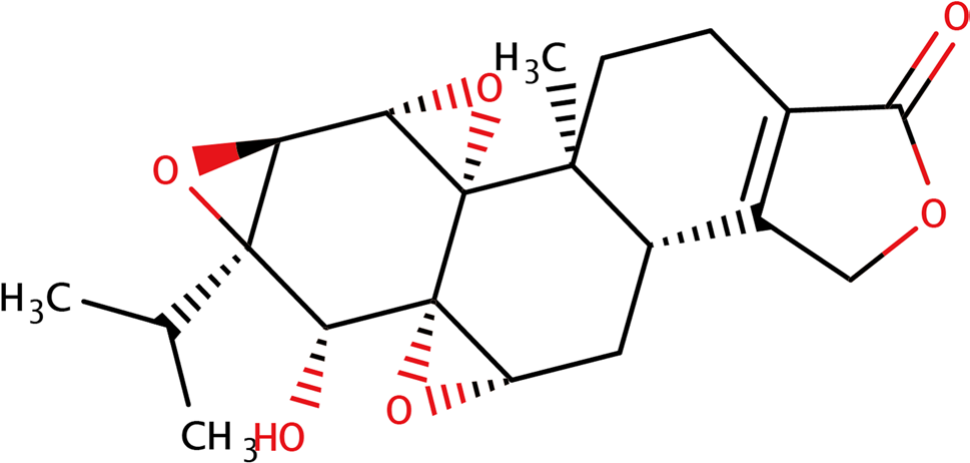
Molecular structure of celastrol

Chinese herbal medicines have been essential sources of drugs for thousands of years. Currently, many medicines are natural products derived from herbs or their derivatives, and these have made significant contributions to the treatment of diseases, especially tumors and infectious diseases [16–18]. Celastrol, the bioactive component of *Tripterygium wilfordii* used in traditional Chinese medicine (Fig. 1), has been shown to have anti-tumor, anti-inflammatory, and immunosuppressive effects [19–21]. Celastrol has anti-tumor properties in many types of cancers, such as melanoma, gastric cancer, breast cancer, glioma, and ovarian cancer [21–25]. As a result, the mechanism underlying celastrol intervention in the occurrence and development of melanoma has become a popular research topic [25, 26].

The cell cycle is a highly regulated and coordinated biological mechanism that ensures the continuous division and precise replication of cells[27]. Simultaneously, aberrations in cell cycle can disrupt the normal cell cycle, constituting a crucial factor in the development of cancer and other diseases[28]. These abnormalities compromise strict control over cell growth, promoting the formation of cancer cells. Cell cycle regulation is also relevant to the treatment of melanoma. Certain treatment modalities, such as cell cycle-specific chemotherapy drugs, can suppress tumor growth and metastasis by interfering with the cell cycle of melanoma cells[29]. Therefore, understanding the role of cell cycle is paramount for unraveling the mechanisms underlying Melanoma pathogenesis.

As a classic signaling pathway, the phosphatidylinositol 3-kinase (PI3K)/AKT/mammalian target of the rapamycin (mTOR) signaling pathway plays a crucial role in the tumorigenesis, proliferation, and progression of human tumors [30, 31]. Notably, inhibition of the PI3K/AKT/mTOR signaling pathway has been shown to lead to effective regression of human tumors [31, 32]. More importantly, targeting the PI3K/AKT/mTOR signaling pathway has demonstrated the ability to inhibit the proliferation and migration of melanoma cells [33, 34]. Furthermore, celastrol can inhibit the growth of B16 melanoma cells and induce apoptotic cell death via activation of the reactive oxygen species (ROS) dependent mitochondrial pathway and suppression of the PI3K/AKT/mTOR signaling [35]. However, the specific mechanism of action needs to be further studied.

Hypoxia is the main characteristic of the TME. Most tumors exhibit various degrees of hypoxia, which is closely associated with clinical prognosis [36]. However, it has been reported that hypoxia-inducible factor 1 alpha (HIF-1α) up-regulation caused by hypoxia in the tumor may be a potential driving force for tumor metastasis [37]. Additionally, studies have shown that increased levels of hypoxia-inducible factors (HIFs) are associated with the progression of various cancers and resistance to radiotherapy and chemotherapy, offering a potential avenue for targeted cancer cell therapy [37–39]. Research has reported that HIF-1α is the downstream transcription factor of mTOR and that the PI3K/AKT/mTOR signaling pathway can maintain the transcription, translation, and biological activity of HIF-1α [40, 41]. Consequently, HIF-1α expression is up-regulated in tumor cells following activation of the PI3K/AKT pathway [42]. Moreover, studies have demonstrated that the PI3K/AKT/mTOR signaling pathway can affect the proliferation and metastasis of melanoma by altering the expression of HIF-1α [40, 43].

However, there is a lack of relevant research investigating how celastrol regulates the expression of HIF-1α and the PI3K/AKT/mTOR signaling pathway to treat B16-F10 melanoma cells, and the precise mechanism involved remains unclear. Thus, further investigation is needed in this area.

In summary, this study demonstrates that the molecular mechanism of celastrol in the treatment of B16-F10 melanoma cells involves the inhibition of HIF-1α mRNA expression by regulating the PI3K/AKT/mTOR signaling pathway.

## Materials and methods

### Antibodies, plasmids, and chemicals

Celastrol (purity of > 99%, cat. No. HY-13067) and PI3K activator (purity of > 99%, cat. No. HY-151527) were purchased from MedChem Express (MCE, shanghai, China) and was dissolved in dimethyl sulfoxide (DMSO) to generate a stock concentration of 10 nM, stored at − 80 °C.p-PI3K (cat. No. 4228), PI3K (cat. No. 4257), p-AKT (cat. No. 4060), AKT (cat. No. 4691), p-mTOR (cat. No. 5536), mTOR (cat. No. 2983), and β-actin (cat. No. 4967) were purchased from Cell Signaling Technology (CST, Woburn, USA). rabbit anti-mouse IgG antibody, and the secondary antibody, goat anti-rabbit IgG antibody was purchased from ZSGB-BIO (Beijing, China). BCA protein assay kit and Cytoplasmic Protein Extraction Kits were purchased from Beyotime (Jiangsu, China).

### Cell culture

C57BL/6 J mice (B16-F10) melanoma cell lines were obtained from the Cell Bank of the Chinese Academy of Sciences (Kunming, China). B16-F10 cells were cultured in Dulbecco's modified Eagle’s medium (DMEM, G4511-500ML, Servicebio, Wuhan, China) supplemented with 10% fetal bovine serum (FBS, 10099-141C, GIBCO, California, USA) and a 1% penicillin/streptomycin solution (CM0001-100ML, Sparkjade, Jinan, China). T25 cm^2^ flasks were used to culture B16-F10 cells at a constant temperature of 37℃ in a moist incubator with 5% CO_2_. The culture medium wasrefreshed every two days.

### Cell viability assay

CCK8 (CT0001-B, Sparkjade, Jinan, China) was used to test the inhibitory effect of celastrol on B16-F10 cell proliferation. B16-F10 cells were seeded into 96-well plates at a density of 6 × 10^3^ cells/well. After overnight incubation, different concentrations (0, 0.03, 0.3, 1, 3, and 10 μM) of celastrol were added to cells for 12, 24, 36, and 48 h, and then cells of each well were treated with CCK-8 solution for 1 h at the end of the incubation period. A wavelength of 450 nm was selected, and an enzyme-linked immunosorbent assay was used to detect the light absorption value of each well.

### Cell clone formation assay

The cells were plated in 6-well plates at a density of 200 cells/well. After 24 h, the cells were treated with different concentrations (0.03, 0.1, 0.3, 1, 3 and 10 μM) of celastrol for 14 days. Subsequently, the medium was discarded, and Cell PBS (1X) was used to rinse the cells three times. Next, colonies were fixed with 4% paraformaldehyde andtreated with 0.1% crystal violet for 15 min, followed by image acquisition using a ZEISS Axio Vert.A1 system.

### Wound healing assay

The effect of various concentrations of celastrol on the migratory ability of B16-F10 cells was evaluated using a wound-healing assay. Approximately 6 × 10^5^ cells were plated in 6-well plates and incubated to 85–95% confluency. A wound was created with a line in the middle using a sterile pipette (200 μl), and cell debris was removed using Cell PBS (1X). Subsequently, various concentrations (0, 0.03, 0.3, 1, 3, and 10 μM) of celastrol were added, and cells were cultured for 0, 12, 24, 36 and 48 h, respectively. Images of wound closure were captured using a ZEISS fluorescence microscope, and subsequent measurements of the wound area were conducted using ImageJ software.

### Flow cytometric analysis of apoptosis

Apoptosis was detected using an Annexin V- FITC Apoptosis Assay Kit (Abs50001, Absin, Shanghai, China). B16-F10 cells were seeded into 24-well plates at a density of 9 × 10^4^ cells/well. The cells were digested using 300 μl of trypsin (CN0004-100ML, Sparkjade, Jinan, China), and then treated with different concentrations of celastrol (0.03, 0.1, 0.3, 1, 3, and 10 μM). After 24 h of treatment, B16-F10 cells were harvested by centrifuging at 1000 rpm for 10 min at 4℃. The cells were then resuspended in 1 ml Cell PBS (1X) and centrifuged repeatedly. Subsequently,300 μl Blinding Buffer (1X) and 5 μl Annexin V-FITC were respectively added to the suspended cells and mixed.The suspended cells were then treated with 5 μl PI for staining after 15 min in the dark. Finally, 200 μl of Blinding Buffer (1X) was added. Assays were conducted using a BD FACSVerse flow cytometer (BD Biosciences).

### Mitochondrial membrane potential assay

A JC-1 Mitochondrial Membrane Potential Assay Kit (C2006, Beyotime, Jiangsu, China) was used for mitochondrial membrane potential (MMP) detection. B16-F10 cells were plated in 6-well plates (6 × 10^5^ cells/well), and various concentrations of celastrol (0, 0.03, 0.3, 1, 3, and 10 μM) were added to respective wells. After 24 h, the cells were harvested, 500 μl of JC-1 staining working solution was added, and the cells were incubated in a CO_2_ incubator at 37 °C for 20 min. JC-1 staining buffer (1X) was used to rinse the cells twice before adding 2 ml DMEM during the final period. The results were analyzed using a BD FACSVerse flow cytometer.

### Detection of reactive oxygen species

B16-F10 cells were plated in 6-well plates (6 × 10^5^ cells/well). Different concentrations of celastrol (0, 0.03, 0.3, 1, 3, and 10 μM) were added to the B16-F10 cells. After 24 h of treatment, the cells were harvested and cultured with 1 ml DCFH-DA (10 μM/L) in a cell incubator for 20 min at 37 °C (S0033S, Beyotime, Jiangsu, China). Subsequently, the cells were rinsed three times with 1 ml DMEM culture medium. A BD FACSVerse flow cytometer was used to perform all assays.

### Western blot analysis

After being incubated in T25 cm^2^ flasks and treated with 10 μM concentration of celastrol for 24 h, B16-F10 cells were lysed with cold RIPA solution. According to the standard protocol,proteins were extracted using a BCA protein assay kit (P0012S, Beyotime, Jiangsu, China) to determine protein concentration. A total of 40 μg of protein was separated using 10–12% SDS-PAGE, transferred onto a PVDF membrane, and cultured with a closed buffer (1% BSA in TBST) for 1 h. The primary antibodies, including p-PI3K (1:1000), PI3K (1:1000), p-AKT (1:1000), AKT (1:1000), p-mTOR (1:1000), mTOR (1:1000), and β-actin (1:1500), were respectively incubated overnight at 4℃ after the membranes were cropped based on the expression levels of the target proteins. Subsequently, the membrane was cultured with the primary antibody, rabbit anti-mouse IgG antibody, and the secondary antibody, goat anti-rabbit IgG antibody (1:15000). This incubation was maintained at 37 °C for 1 h. The membranes were then observed with an enhanced chemiluminescence detection system (Fusion FX7, Vilber, France) and quantified using ImageJ software.

### Cell cycle assay

B16-F10 cells were plated at a density of 9 × 10^5^ cells/well in 24-well plates. The cells were digested using 300 μl of trypsin and then treated with different concentrations of celastrol.Next, the cells were fixed overnight in 70% ethanol. Cells were then harvested by centrifugation at 1000 rpm for 10 min at 4 °C and rinsed with 1 ml Cell PBS (1X). Subsequently, cells were incubated with staining buffer, RNase A configuration propidium iodide staining working solution (C1052, Beyotime, Jiangsu, China), and 0.5 ml propidium iodide staining working solution (20X) for 30 min at 37 °C in a cell incubator. Samples were examined using a BD FACSVerse flow cytometer.

### Reverse transcription-quantitative (RT-q) PCR assay

RNA was extracted from B16-F10 cells treated with different concentrations of celastrol using the SPARKeasy Tissue/Cell RNA Rapid Extraction Kit according to the manufacturer’s instructions (AC0202, Sparkjade, Jinan, China). Reverse transcription was conducted using the HiScript II Q Select RT SuperMix for qPCR (R233-01 and Q711-02/03, Vazyme, Nanjing, China). A reaction volume of 20 μl was used for HIF-α analysis, with GAPDH as an internal control to standardize the expression levels of HIF-α. The relative quantitative analysis was conducted using the 2-ΔΔCt method.

The HIF-α primers used were 5'-CAAGTCAGCAACGTGGAAGG-3' (forward) and 5'-ATCAGCACCAAGCACGTCAT-3' (reverse).

The GAPDH primers used were 5'-CCTCGTCCCGTAGACAAAATG-3' (forward) and 5'-TGAGGTCAATGAAGGGGTCGT-3' (reverse).

### Statistical analysis

Data were analyzed by one-way analysis of variance (ANOVA) using GraphPad Prism version 8.0.1 statistical software and were presented as mean ± SD. *P* < 0.05 was considered statistically significant.

## Results

### Celastrol inhibited B16-F10 cell proliferation and cell cloning

**Fig. 2:**
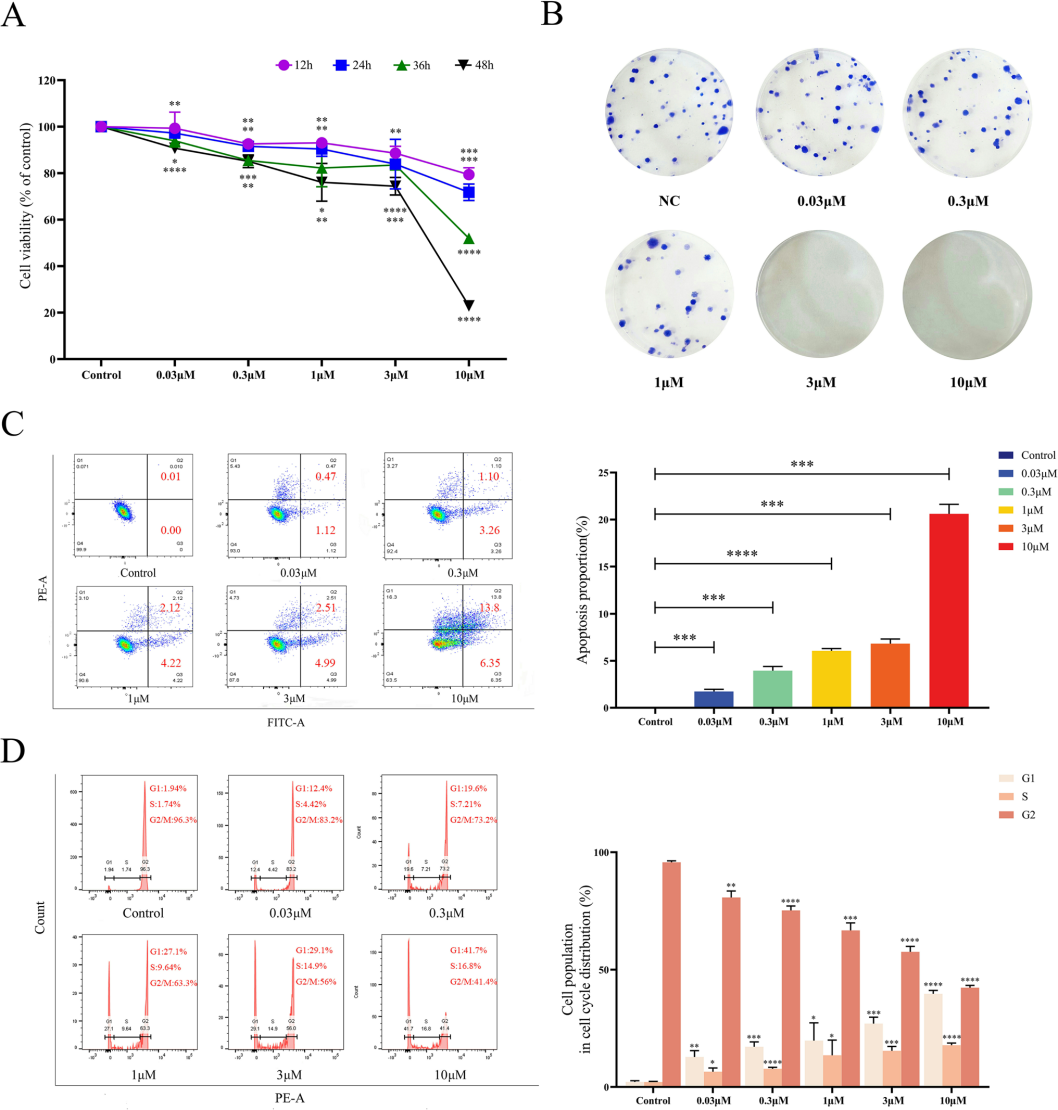
The inhibitory effect of celastrol on the cell viability, cell apoptosis, and cell cycle of B16-F10 cells. **A** B16-F10 cell viability was inhibited by different concentrations of celastrol (0, 0.03, 0.3, 1, 3, and 10 μM) at 12, 24, 36, and 48 h, as assessed using a CCK8 assay. **B** B16-F10 cells were treated with different concentrations of celastrol for 14 days, and cell colony formation was evaluated using a clone-formation assay. **C** B16-F10 cells were treated with different concentrations of celastrol, and the apoptotic cells were analyzed by flow cytometry using an Annexin V-FITC/PI kit. **D** B16-F10 cells were treated with different concentrations of celastrol for 24 h, and the cell cycle distribution was analyzed using flow cytometry. (Data are presented as the mean ± SD, n = 4. ^*^P < 0.05, ^**^P < 0.01, ^***^P < 0.001, ^****^P < 0.0001 compared to control group). The results represent one set of findings from three independent replicates

This study aimed to investigate the inhibitory effects of celastrol on the growth of B16-F10 cells. B16-F10 cells were treated with different concentrations of celastrol (0, 0.03, 0.3, 1, 3, and 10 μM) for 12, 24, 36, and 48 h, and cell viability was assessed using a CCK8 assay (Fig. 2A). We found that celastrol exerted a time-and dose-dependent inhibition on the viability of B16-F10 cells, with the most significant cell death observed at a concentration of 10 µM. To further demonstrate the inhibitory effects of celastrol on B16-F10 cells, the cells were treated with different concentrations of celastrol for 14 days, and the number of colonies formed was counted (> 50 colonies) (Fig. 2B). The results showed that celastrol inhibited the formation of B16-F10 cell colonies in a concentration-dependent manner, with the most pronounced cell death observed at concentrations of 3 µM and 10 µM.

### Celastrol induced B16-F10 cell apoptosis and B16-F10 cell cycle arrest

B16-F10 cells were treated with different concentrations of celastrol (0, 0.03, 0.3, 1, 3, and 10 μM) for 24 h, and the apoptosis rates of the cells were detected to explore the effect of celastrol on B16-F10 cell apoptosis. The results showed that the apoptosis rate of B16-F10 cells gradually increased with increasing celastrol concentration (Fig. 2C).

The effect of celastrol on the cell cycle was determined using the aforementioned culture method. The results showed that celastrol affected the cell cycle of B16-F10 cells, increased the number of cells in the G1 and S phases, and decreased the number of cells in the G2/M phase (Fig. 2D). These findings indicated that celastrol inhibited the cell cycle transition from the G1 and S phases to the G2/M phase, which was directly related to the apoptosis of B16-F10 cells.

### Celastrol inhibited the migration of B16-F10 cells

**Fig. 3:**
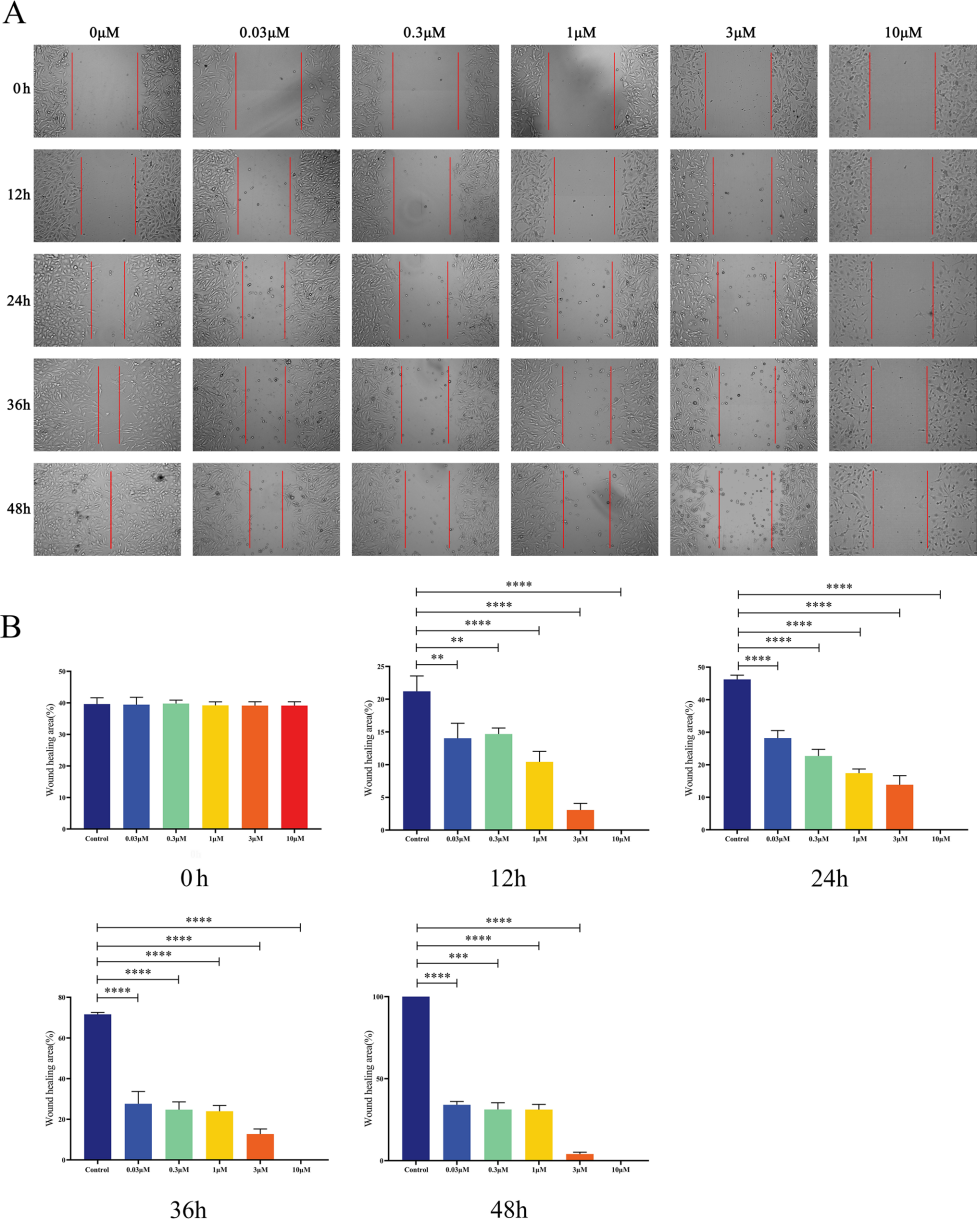
The effect of celastrol on the migration of B16-F10 cells. **A** After creating a wound in the middle of the well, B16-F10 cells were treated with increasing concentrations of celastrol. **B** Cell migratory capacity was assessed at 12, 24, 36, and 48 h by calculating the wound closure area. (Data are presented as the mean ± SD, n = 4. ^**^P < 0.01, ^***^P < 0.001, ^****^P < 0.0001 compared to control group). The results represent one set of findings from three independent replicates

We investigated the effects of celastrol at different concentrations on the migration of B16-F10 cells at different time points using a microscope. Celastrol at concentrations of 3 and 10 μM demonstrated the most significant impact on the wound healing of B16-F10 cells. These results confirmed that celastrol inhibited the migration and proliferation of B16-F10 cells in a dose-dependent manner (Fig. 3A, B).

### Celastrol increased intracellular ROS levels and decreased the mitochondrial membrane potential in B16-F10 cells

**Fig. 4:**
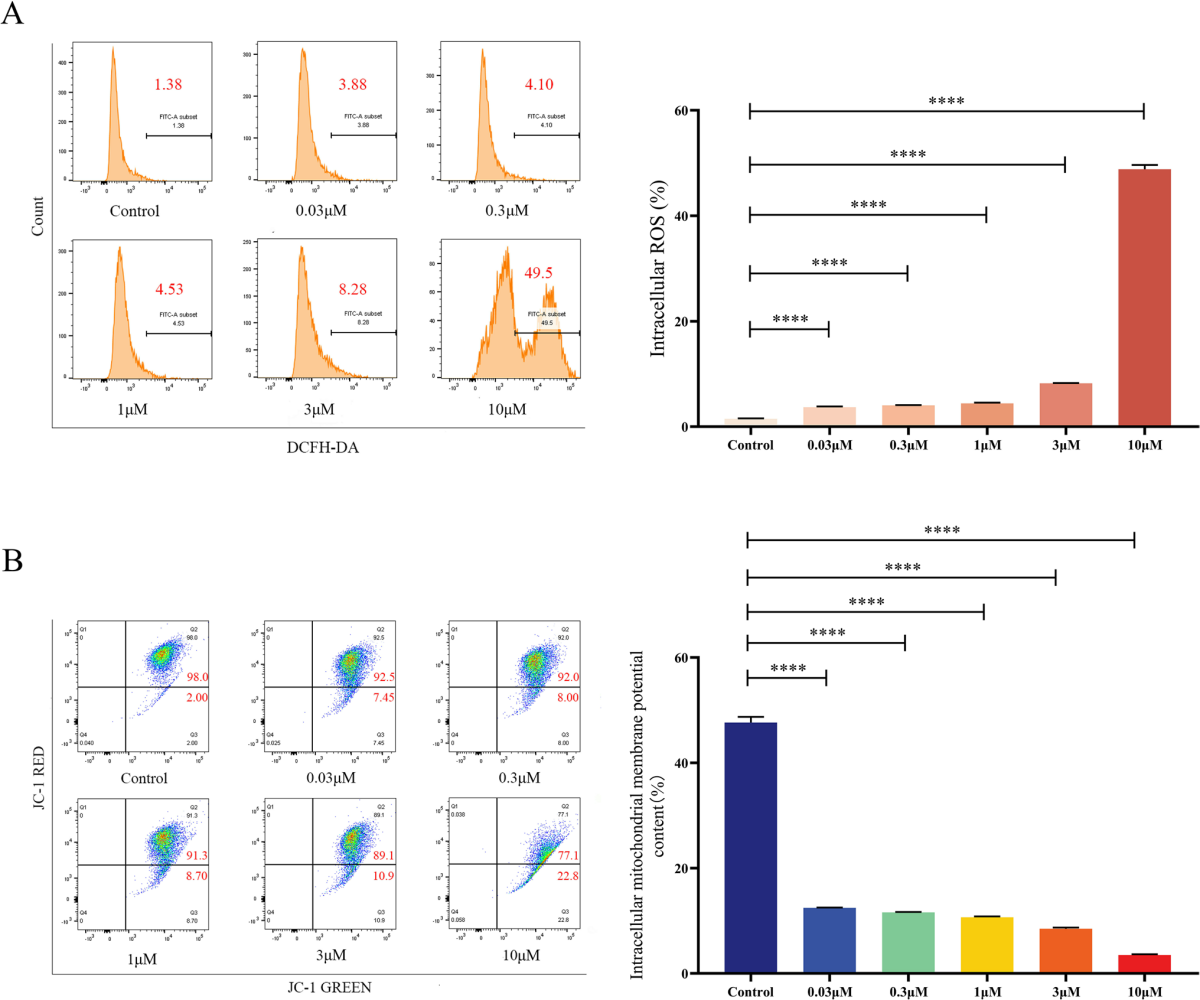
Celastrol induced ROS accumulation and decreased the mitochondrial membrane potential in B16-F10 cells. **A** B16-F10 cells were treated with different concentrations of celastrol (0, 0.03, 0.3, 1, 3, and 10 μM) for 24 h and stained with DCFH-DA, and the intracellular ROS content was detected using flow cytometry. **B** B16-F10 cells were treated with different concentrations of celastrol for 24 h and stained with JC-1 staining solution, and the intracellular ratio of the B16-F10 cells was determined using flow cytometry. (Data are presented as the mean ± SD, n = 4. ^****^P < 0.0001 compared to control group). The results represent one set of findings from three independent replicates

To demonstrate that celastrol could induce an increase in ROS levels in B16-F10 cells, B16-F10 cells were treated with different concentrations of celastrol (0, 0.03, 0.3, 1, 3, and 10 μM) for 24 h and stained with DCFH-DA, and the intracellular ROS content was detected by flow cytometry. The results showed that the ROS content increased with increasing celastrol concentrations in a dose-dependent manner (Fig. 4A).

To confirm the effect of celastrol on the mitochondrial membrane potential of B16-F10 cells, the experimental conditions were kept consistent with the earlier findings. The results revealed that the mitochondrial membrane potential declined in a dose-dependent manner with increasing celastrol concentrations, particularly at the 3 and 10 μM concentrations. This difference was statistically significant (Fig. 4B).

### Celastrol restrained the PI3K/AKT/mTOR pathway in B16-F10 cells, resulting in decreased expression of HIF-α mRNA

**Fig. 5:**
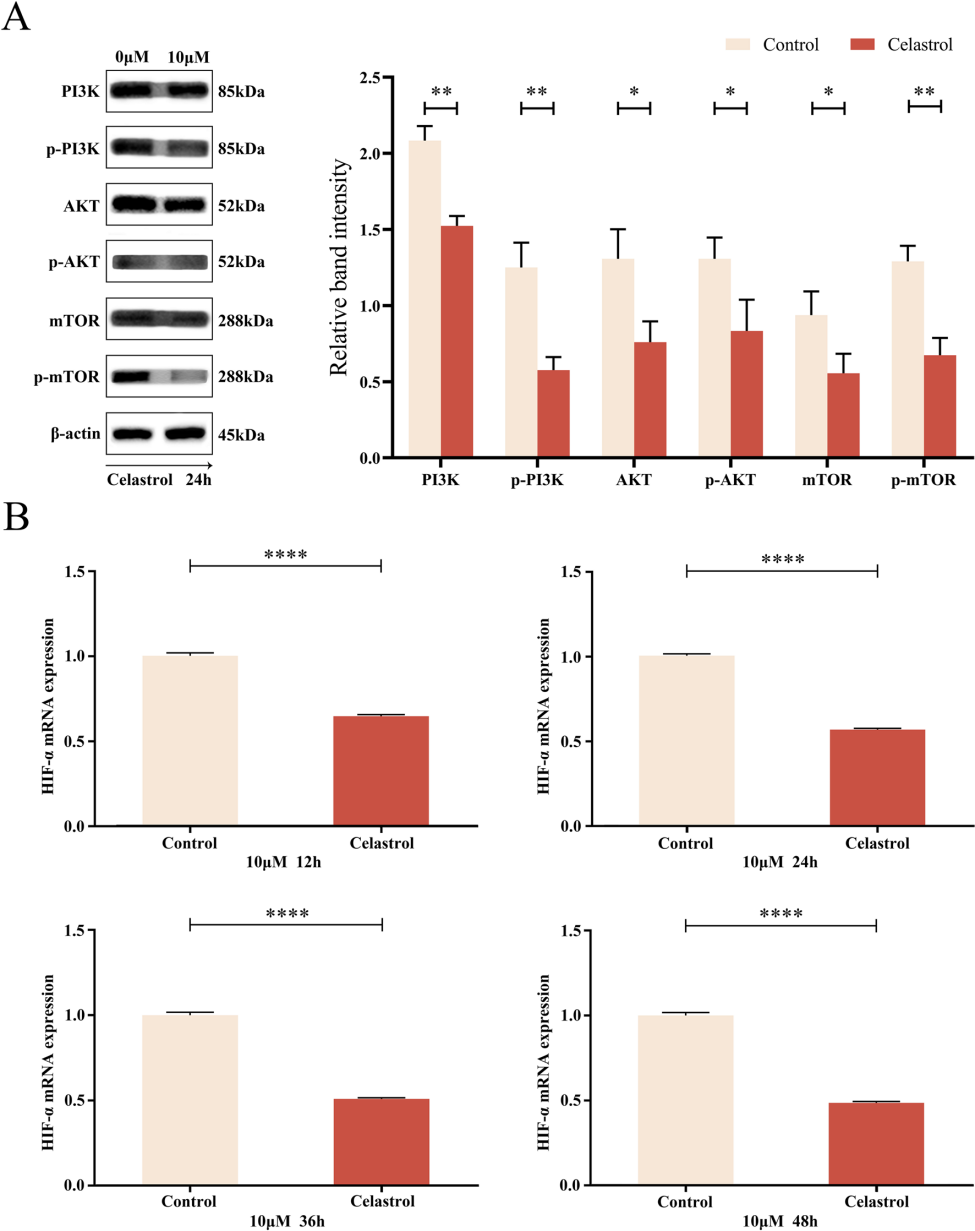
Celastrol inhibited the PI3K/AKT/mTOR pathway in B16-F10 cells and led to a decrease in HIF-α mRNA expression. **A** B16-F10 cells were treated with 10 μM celastrol for 24 h, and the levels of p-PI3K, PI3K, p-AKT, AKT, p-mTOR, and mTOR were detected by western blotting. **B** After B16-F10 cells were treated with 10 μM celastrol for 12, 24, 36, and 48 h, the expression of HIF-α mRNA was detected by qPCR. (Data are presented as the mean ± SD, n = 3. ^*^P < 0.05, ^**^P < 0.01, ^****^P < 0.0001 compared to control group). The results reprent one set of findings from three independent replicates

To investigate the molecular pathway of B16-F10 cells in response to incubation with 10 μM celastrol,the levels of PI3K, AKT and mTOR were detected after 24 h. The western blot assay results demonstrated that celastrol significantly inhibited PI3K, AKT, and mTOR expression and phosphorylation (Fig. 5A).

After the cells were incubated with 10 μM celastrol for 12, 24, 36, and 48 h, the HIF-α mRNA expression in B16-F10 cells was detected by qPCR. The obtained results showed that celastrol inhibited HIF-α mRNA expression at different time points. These findings strongly suggest that celastrol could inhibit the PI3K/AKT/mTOR pathway in B16-F10 cells, leading to a decrease in HIF-α mRNA expression (Fig. 5B).

### Activation of PI3K suppressed the effect of celastrol on B16-F10 cells

**Fig. 6:**
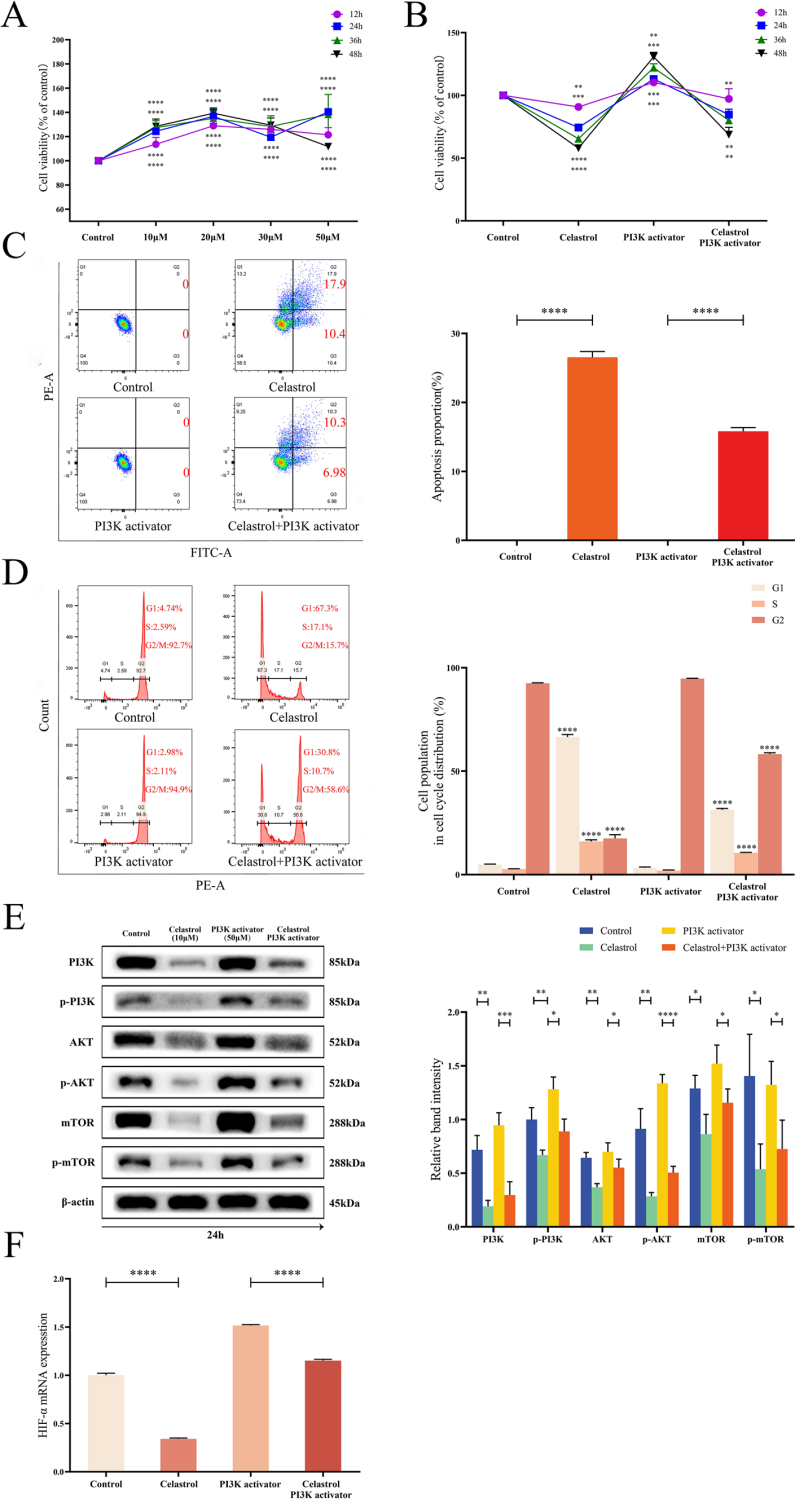
The PI3K activator inhibited the effect of celastrol on B16-F10 cells. **A** B16-F10 cells were treated with different concentrations of PI3K activator (0, 10, 20, 30, and 50 μM) at 12, 24, 36, and 48 h, and the cell viability was detected using a CCK8 assay. Subsequently, 50 μM PI3K activator and 10 μM celastrol were selected for co-culture for 24 h for subsequent experiments. **B** Cells were co-cultured in celastrol and PI3K activator as described above, and a CCK8 assay was used to detect cell viability. **C** The apoptosis rate of B16-F10 cells was detected by flow cytometry under the same conditions. **D** The cell cycle was evaluated by flow cytometry under the same conditions. **E** The levels of p-PI3K, PI3K, p-AKT, AKT, p-mTOR, and mTOR were detected using western blotting under the same conditions. **F** The expression of HIF-α mRNA in B16-F10 cells was detected by qPCR after co-culture with celastrol and PI3K activator. (Data are presented as the mean ± SD, n = 4. ^*^P < 0.05, ^**^P < 0.01, ^****^P < 0.0001 compared to control and ^*^P < 0.05, ^***^P < 0.001, ^****^P < 0.0001 compared to PI3K activator group). The results represent one set of findings from three independent replicates

The therapeutic effects of celastrol were further verified by incubating B16-F10 cells with a PI3K activator (compound AE-18). B16-F10 cells were treated with different concentrations of the PI3K activator (0, 10, 20, 30, and 50 μM) for 12, 24, 36, and 48 h, and the cell viability was measured using a CCK8 assay. The results showed that the PI3K activator enhanced the cell viability of B16-F10 cells in a concentration-dependent manner, and the 50 μM PI3K activator promoted the cell viability of B16-F10 cells most obviously at 24 h (Fig. 6A). Therefore, after co-cultivating B16-F10 cells with 50 μM PI3K activator and 10 μM celastrol for 24 h for subsequent experiments, the results showed that the PI3K activator inhibited the inhibitory effect of celastrol on the viability of B16-F10 cells (Fig. 6B).

Apoptosis and cell cycle progression were detected according to the culture period and concentration. The results of flow cytometry showed that the PI3K activator inhibited the effects of celastrol in promoting the apoptosis of B16-F10 cells and inhibiting the cell cycle (Fig. 6C, D). In addition, western blot analysis of PI3K, AKT, and mTOR protein activation demonstrated that the PI3K activator could increase the protein levels of PI3K, AKT and mTOR, thereby further mitigating the inhibitory effects of celastrol on these proteins. These results corroborate celastrol's inhibition of B16-F10 cells survival through the PI3K/AKT/mTOR pathway (Fig. 6E). Following the use of qPCR to detect the change in HIF-α mRNA expression within B16-F10 cells after co-cultured with PI3K activator, it was observed that the PI3K activator could increase the expression of HIF-α mRNA and further balance the inhibitory effect of celastrol on HIF-α (Fig. 6F).

### The molecular mechanism of celastrol inhibited B16-F10 cell survival via regulating PI3K/AKT/mTOR signaling pathway and repressing HIF-1α mRNA expression

**Fig. 7:**
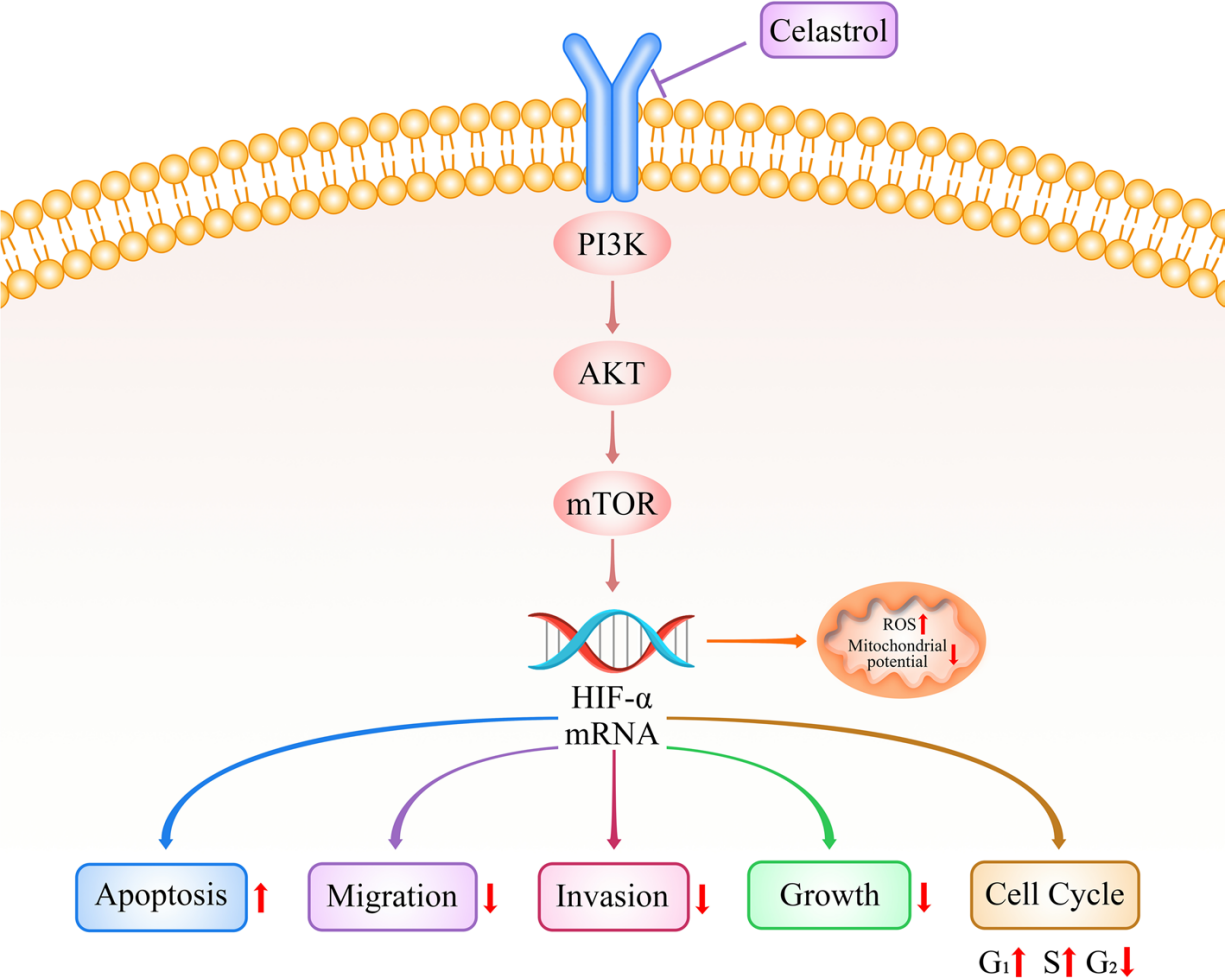
The molecular mechanism of celastrol in inhibiting B16-F10 cells

The results demonstrated that celastrol could induce the downregulation of HIF-α mRNA by inhibiting the PI3K/AKT/mTOR pathway, promoting an increase in ROS levels and a decrease of mitochondrial membrane potential in B16-F10 cells, thereby resulting in the promotion of cell apoptosis and a reduction in cell migration, invasion, and cell proliferation. Additionally, celastrol induced cell cycle arrest by increasing the G1 and S phases and decreasing the G2/M phase (Fig. 7).

## Discussion

This study demonstrated that celastrol affects B16-F10 melanoma cells by inhibiting the PI3K/AKT/mTOR signaling pathway and the expression of HIF-α mRNA, indicating that celastrol inhibits B16-F10 melanoma cell migration and survival. The results also found that celastrol increased ROS and decreased mitochondrial membrane potential, leading to cell cycle arrest and promoting apoptosis. Meanwhile, the activation of PI3K enhanced the migration and invasion abilities of B16-F10 melanoma cells and attenuated the inhibitory effect of celastrol, which further suggests the potential mechanism of action of celastrol in therapeutic strategies targeting B16-F10 melanoma cells.

Currently, melanoma has relatively lower morbidity and mortality rates compared to common cancers such as including breast and lung cancers. However, the prognosis of patients with advanced melanoma is usually poor due to its tendency to metastasize and spread to other organs [7–9]. Therefore, it is essential to identify effective therapeutic drugs that can inhibit the growth and metastasis of melanoma with minimal toxicity.

Celastrol, a natural extract, has been widely studied for the treatment of various tumors and infectious diseases [18–20]. The expression of HIF-1α mRNA may be a potential driving factor for tumor growth and metastasis, and HIF-1α is a downstream transcription factor of mTOR [38]. Previous studies have highlighted that HIF-1α can affect the progression of melanoma through the PI3K/AKT/mTOR signaling pathway, and celastrol has been demonstrated to inhibit the growth of melanoma cells by inhibiting PI3K/AKT signaling [36, 41, 42]. It has also been reported that HIF-1α can affect the regulation of tumor chemokines by miRNA in the B16-F10 melanoma mouse tumor model [44]. However, the molecular mechanism underlying celastrol-induced apoptosis in melanoma cells has not been fully elucidated.

Our findings corroborate previous studies showing celastrol's inhibitory effects on melanoma cell proliferation, viability, and tumor growth upon inhibition of PI3K and mTOR [2, 3]. Additionally, some studies have shown that mTOR inhibition is attenuated in melanoma cells due to a feedback mechanism that may promote AKT activation [40]. In addition, mTOR activation appears to be independent of AKT signaling in other melanoma cell lines [42, 43]. However, our study demonstrated a correlation between mTOR and AKT expression, with AKT and mTOR expression being downregulated following PI3K activation.

Previous studies have presented conflicting results regarding the impact of ROS levels in tumor cells. For instance, some research reports suggest that compounds such as celastrol induce apoptosis of tumor cells by increasing ROS levels [21, 23]. However, it is important to note that excessive ROS levels also damage the normal function of important cells and promote tumor occurrence and chemotherapy resistance [4]. In our study, we observed that celastrol promoted ROS production in a dose-dependent manner during our investigation of ROS levels.

Overall, our results indicate the promising potential of celastrol for the prevention of B16-F10 melanoma cell progression. By affecting the PI3K/AKT/mTOR pathway and the expression of the downstream transcription factor HIF-α, which play vital roles in tumors, celastrol has demonstrated its ability to impede the growth of B16-F10 melanoma cells.

The innovative aspect of our study is centered around showcasing the therapeutic effect of celastrol, particularly in the B16-F10 melanoma cell line. Moreover, we sought to validate its influence on HIF-α, a downstream transcription factor of mTOR, by examining the PI3K/AKT/mTOR pathway, which has not been explored in previous research. This led us to investigate both the therapeutic effects and the mechanism of action of celastrol on B16-F10 cells.

This study has some limitations that should be considered. For instance, the study did not examine alternate melanoma cell lines to verify the therapeutic effect of celastrol or further verify the effect of celastrol in mouse models of melanoma. Additionally, applying more cutting-edge experimental techniques for verification could enhance the study's quality and comprehensiveness. Furthermore, it is worth noting that some reports suggest miRNA's potential role in regulating PI3K/AKT/mTOR and HIF-α in other tumors [45–47]. Therefore, our future research will explore the mechanism of celastrol in treating B16-F10 melanoma at the molecular level of miRNAs.

## Conclusions

Our results demonstrate that celastrol effectively inhibits B16-F10 melanoma cell survival. Importantly, this effect is achieved through the downregulation of HIF-α mRNA by inhibiting the PI3K/AKT/mTOR pathway. Additionally, celastrol promotes an increase in ROS and a decrease in mitochondrial membrane potential in B16-F10 melanoma cells, resulting in enhanced cell apoptosis, reduced cell migration, invasion, and proliferation, as well as changes in the cell cycle. These results illustrate themechanism of action of celastrol in melanoma and provide a potential reference for clinical research.

## Data Availability

Data in this article can be reasonably requested from the corresponding author.
